# Endothelial siRNA delivery in nonhuman primates using ionizable low–molecular weight polymeric nanoparticles

**DOI:** 10.1126/sciadv.aar8409

**Published:** 2018-06-27

**Authors:** Omar F. Khan, Piotr S. Kowalski, Joshua C. Doloff, Jonathan K. Tsosie, Vasudevan Bakthavatchalu, Caroline Bodi Winn, Jennifer Haupt, Morgan Jamiel, Robert Langer, Daniel G. Anderson

**Affiliations:** 1 David H. Koch Institute for Integrative Cancer Research, Massachusetts Institute of Technology, 500 Main Street, Cambridge, MA 02139, USA.; 2 Department of Chemical Engineering, Massachusetts Institute of Technology, 77 Massachusetts Avenue, Cambridge, MA 02139, USA.; 3 Department of Anesthesiology, Boston Children’s Hospital, 300 Longwood Avenue, Boston, MA 02115, USA.; 4 Division of Comparative Medicine, Massachusetts Institute of Technology, Cambridge, MA 02139, USA 02139.; 5 Division of Health Science Technology, Massachusetts Institute of Technology, MA 02139, USA.; 6 Institute for Medical Engineering and Science, Massachusetts Institute of Technology, Cambridge, MA 02139, USA.

## Abstract

Dysfunctional endothelial cells contribute to the pathophysiology of many diseases, including vascular disease, stroke, hypertension, atherosclerosis, organ failure, diabetes, retinopathy, and cancer. Toward the goal of creating a new RNA-based therapy to correct aberrant endothelial cell gene expression in humans, efficient gene silencing in the endothelium of nonhuman primates was achieved by delivering small interfering RNA (siRNA) with 7C1, a low–molecular weight, ionizable polymer that forms nanoparticles. After a single intravenous administration of 1 mg of siRNA per kilogram of animal, 7C1 nanoparticles delivering Tie2 siRNA caused Tie2 mRNA levels to decrease by approximately 80% in the endothelium of the lung. Significant decreases in Tie2 mRNA were also found in the heart, retina, kidney, pancreas, and bone. Blood chemistry and liver function analysis before and after treatment all showed protein and enzyme concentrations within the normal reference ranges. Furthermore, after controlling for siRNA-specific effects, no significant increases in inflammatory cytokine concentrations were found in the serum. Similarly, no gross lesions or significant underlying pathologies were observed after histological examination of nonhuman primate tissues. This study is the first demonstration of endothelial gene silencing in multiple nonhuman primate organs using systemically administered siRNA nanoparticles and highlights the potential of this approach for the treatment of disease in humans.

## INTRODUCTION

Healthy endothelial cells are essential for important physiological functions, including hemostasis, immune response, tissue growth, and vascular regulation. However, dysfunctional endothelium is a hallmark of many diseases, including peripheral vascular disease, stroke, hypertension, atherosclerosis, diabetes, organ failure, certain types of infections, and cancer (*1*). For many of these diseases, an effective endothelial cell–focused therapeutic could improve patient outcomes. One such method is RNA interference (RNAi), a technique that uses small interfering RNA (siRNA) (*2*, *3*). RNAi can be used to silence aberrant gene expression in the endothelium; however, to fully realize the therapeutic potential of this type of intervention, one must first ensure the preferential delivery of siRNA to the endothelium (*4*). While several recent reports have showcased the development of nanoparticles capable of potent, efficient siRNA delivery in nonhuman primates (*5*–*7*) and humans (*8*) after systemic administration, these nanoparticles primarily deliver siRNA to hepatocytes and myeloid cells, not endothelial cells. Patisiran, an siRNA nanoparticle, completed an APOLLO phase 3 trial and appears to be on track for U.S. Food and Drug Administration approval, but it is for hepatic gene silencing (*8*–*10*). Aganirsen (GS-101), a naked modified siRNA, does target the IRS-1 gene in endothelial cells, which is overexpressed in neovascular glaucoma. It is currently in phase 2/3 clinical trials (ClinicalTrials.gov identifier NCT02947867) (*11*). However, aganirsen is administered via eye drops and not systemically via nanoparticles. Atu027, a liposomal siRNA formulation targeting protein kinase N3, has shown RNAi in lung endothelium after five doses in nonhuman primates; however, efficacy in the endothelium of additional organs was not explored (*12*). To the best of the our knowledge, there are no nanoparticle systems capable of achieving preferential delivery of siRNA to the endothelium of multiple nonhuman primate organs (including lung, heart, kidney, and retina) after systemic administration.

**Fig. 1:**
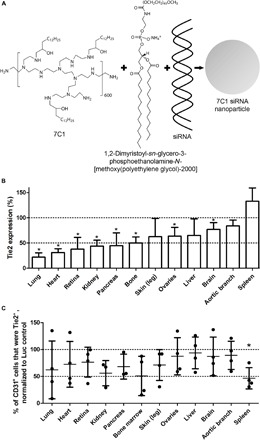
7C1 description and in vivo efficacy in nonhuman primates. (**A**) Schematic representation of 7C1 siRNA nanoparticles. An example chemical structure of a 7C1 repeat unit is shown. 7C1 is mixed in a microfluidic device with a lipid poly(ethylene glycol) and siRNA to form the final nanoparticles. Refer to fig. S1B for full nanoparticle characterization. (**B**) Tie2 mRNA levels in nonhuman primate tissues 48 hours after the intravenous infusion of 7C1-Tie2 siRNA nanoparticles at 1 mg/kg dose, as measured by QuantiGene 2.0 assay. Tie2 mRNA levels were normalized to those measured in nonhuman primates treated with a corresponding dose of luciferase (Luc) control siRNA. Error bars are +SD, and * indicates a significant change (*P* < 0.05, *t* test). (**C**) Percentage of CD31^+^ (endothelial) cells that were also Tie2^+^. The percentages from Tie2 siRNA treatments were normalized to those of Luc control siRNA treatments. Error bars are ±SD, and * indicates a significant change (*P* < 0.05, *t* test) in Tie2 protein levels after Tie2 siRNA treatment, as compared to Luc control siRNA treatments. Note that organs along the *x* axis are arranged to match the order found in (B).

To overcome this challenge, an ionizable, low–molecular weight polymer, 7C1, was developed (*13*). 7C1 complexed with siRNA and a lipid poly(ethylene glycol), the latter of which aids with self-assembly, forms stable nanoparticles (Fig. 1A). These nanoparticles, which have 7C1/lipid poly(ethylene glycol)/siRNA mass ratio of 1:0.19:5, preferentially deliver siRNA to the endothelium after intravenous infusion (*13*, *14*). These 7C1 siRNA nanoparticles have been used in many rodent models of vascular disease and cancer, targeting genes involved in angiogenic signaling [VEGFR-2 (vascular endothelial growth factor receptor–2), Tie1/2, and DLL4], vascular permeability (VE-cadherin), inflammation (ICAM-1/2, VCAM-1, E-selectin, and P-selectin), transcription factors (SOX2, OLIG2, SALL2, and POU3F2), and microRNA (*13*, *15*–*21*). As the next stage of 7C1 development, new bridging studies to transition from small animal models to more human-like species are required. These studies serve as an early foundation for a potential investigational new drug application. Toward the goal of achieving 7C1 clinical translation, the utility of this formulation in larger human-like animal models was evaluated through an examination of its performance in nonhuman primates.

## RESULTS

### 7C1 and siRNA validation and characterization

To test the ability of 7C1 nanoparticles to achieve effective gene silencing in the endothelium, we selected Tie2 as the target gene because of its putative endothelial cell specificity (*22*–*25*). In tumors, Tie2 can be highly overexpressed and found outside the vascular compartment (*26*–*28*), although the animals used in these studies were healthy. After confirming sequence homology of the Tie2 siRNA target site in mouse and nonhuman primate, the efficacy of the Tie2 siRNA formulated in 7C1 nanoparticles was first analyzed in a dose-response study in mice (fig. S1A). The particle sizes measured by dynamic light scattering of the Tie2 and Luc control 7C1 siRNA nanoparticles were 86.8 ± 2.6 nm and 109.6 ± 2.6 nm, respectively. Figure S1 (B and C) shows the particle size distributions.

### Tie2 mRNA silencing in nonhuman primates

For nonhuman primate studies, a dose of Tie2 siRNA (1 mg/kg) was selected to ensure sufficient signal-to-noise resolution in Tie2 transcript knockdown. As a control, a dose of Luc siRNA (1 mg/kg) formulated in 7C1 nanoparticles was used. The sequences of both siRNAs are listed in Materials and Methods. 7C1 siRNA nanoparticles diluted in phosphate-buffered saline (PBS) were infused intravenously into nonhuman primates. No symptoms of infusion reactions were observed after administration of the test articles. Two days later, the animals were euthanized, and their tissues were harvested for protein and mRNA analysis.

Tie2 mRNA levels were examined in the treatment (Tie2 siRNA) and control (Luc control siRNA) groups. Compared to controls, Tie2 knockdown was found in multiple tissues, with the lung and heart showing the greatest reduction (Fig. 1B). No statistically significant changes in Tie2 mRNA were found in the liver, aortic branch, and spleen (*P* > 0.05, *t* test).

### Tie2 protein levels

The amounts of Tie2 protein present in the endothelium of each tissue type were determined by flow cytometry. Tissues were dissociated into single-cell suspensions using a gentleMACS Dissociator (Miltenyi Biotec) and stained for the presence of the CD31 and Tie2 markers. The Tie2 levels in the CD31^+^ endothelial cell population were then measured.

As shown in Fig. 1C and fig. S2A, endothelial cells were identified by the CD31 marker (*29*). Then, the percentage of those CD31^+^ cells that expressed Tie2 was quantified. The percentage values calculated for the Tie2 siRNA treatments were then normalized to the percentage values calculated for the Luc control siRNA treatments. While CD31^+^ was used to gain additional resolution by preventing nonendothelial cells from contaminating the analysis, it can still be variably expressed by other cell types (*30*). Figure S2A shows the gating strategy used to help mitigate these challenges, although follow-on studies with more comprehensive cellular population analyses are planned for the future.

In contrast to mRNAdata sets, larger intra-animal variability in the Tie2 protein expression data (Fig. 1C) was observed. The variability suggests that 48 hours is a suboptimal time point for the analysis of protein levels (an additional time course experiment is recommended to determine the optimal time point in future studies). In addition, the Tie2 median fluorescence intensities (MFIs) within the CD31^+^ population were analyzed (fig. S2B), showing minor MFI changes in the kidney and spleen following Tie2 siRNA treatment (*P* < 0.05, *t* test).

### Blood chemistry and liver function tests

**Fig. 2:**
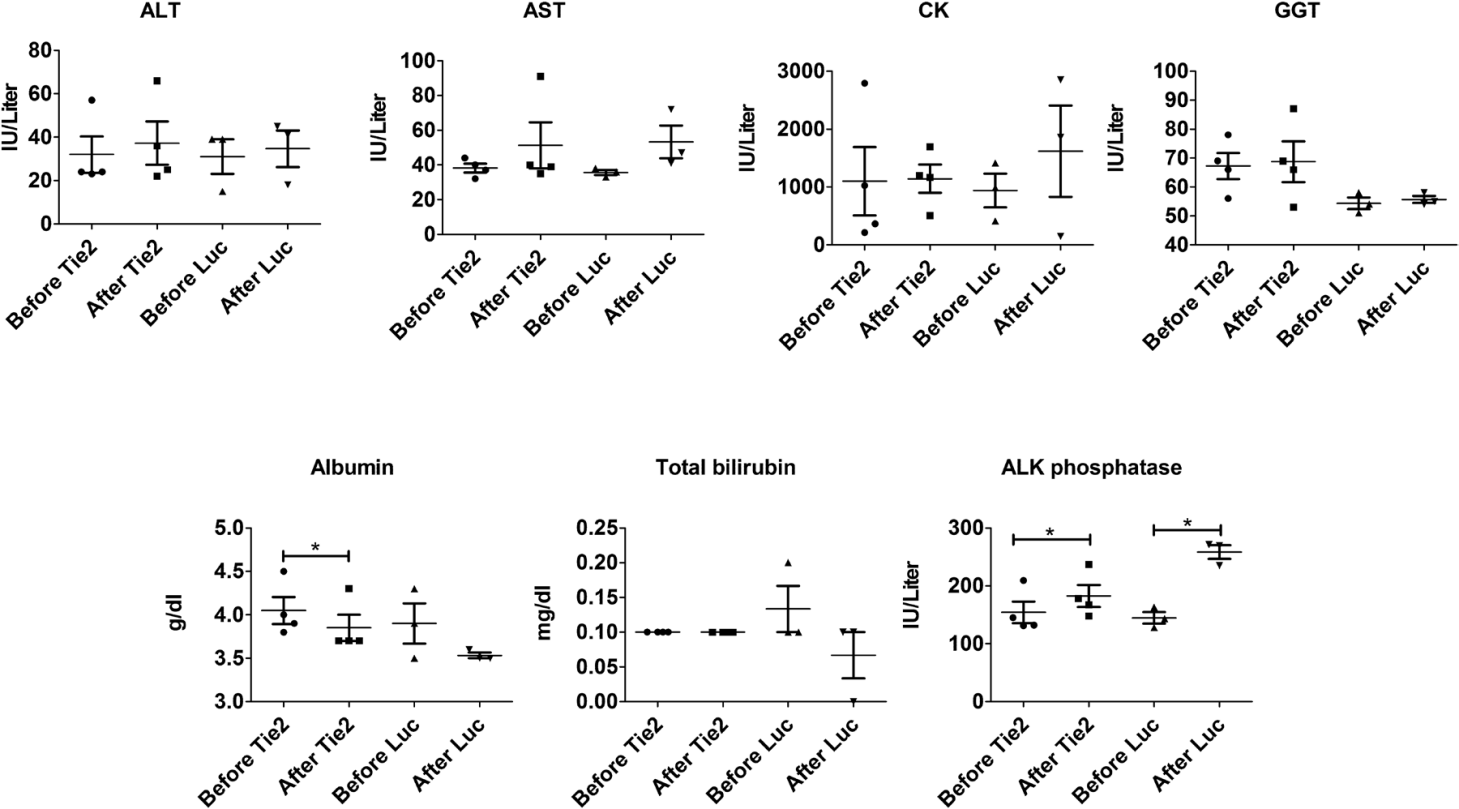
Nonhuman primate blood chemistry and liver function data before and after 7C1 siRNA treatment. Levels were examined for both Tie2 and Luc control siRNA treatments. A *t* test was used to determine any temporal changes in concentrations, and horizontal lines with an * above indicate *P* < 0.05. Regardless of statistically significant differences, all measured concentrations fell within their respective normal reference ranges.

Using a paired experiment setup, blood samples from nonhuman primates before and after treatment were collected for blood chemistry analysis and liver function tests to gain some preliminary insight into the safety and tolerability of the nanoparticle formulations. To control for the specific effects of Tie2 siRNA treatment, we also analyzed samples treated with Luc control siRNA 7C1 nanoparticles. We assessed liver function by alkaline (ALK) phosphatase, alanine aminotransferase (ALT), aspartate aminotransferase (AST), γ-glutamyl transpeptidase (GGT), albumin, and total bilirubin. In addition, creatine kinase (CK) was measured to assess potential muscle inflammation and damage. As seen in Fig. 2, an increase in ALK phosphatase after treatment with both Tie2 and Luc control siRNAs was observed (*P* < 0.05, *t* test). In addition, we found a decrease in albumin after Tie2 siRNA treatment (*P* < 0.05, *t* test), although we observed no corresponding change with Luc control siRNA (*P* = 0.26, *t* test). Nevertheless, we found all measured pre- and posttreatment levels to be within their normal physiological range, indicating that treatment with these nanoparticle formulations did not significantly perturb liver function under these experimental conditions.

### Inflammatory cytokine screen

**Fig. 3:**
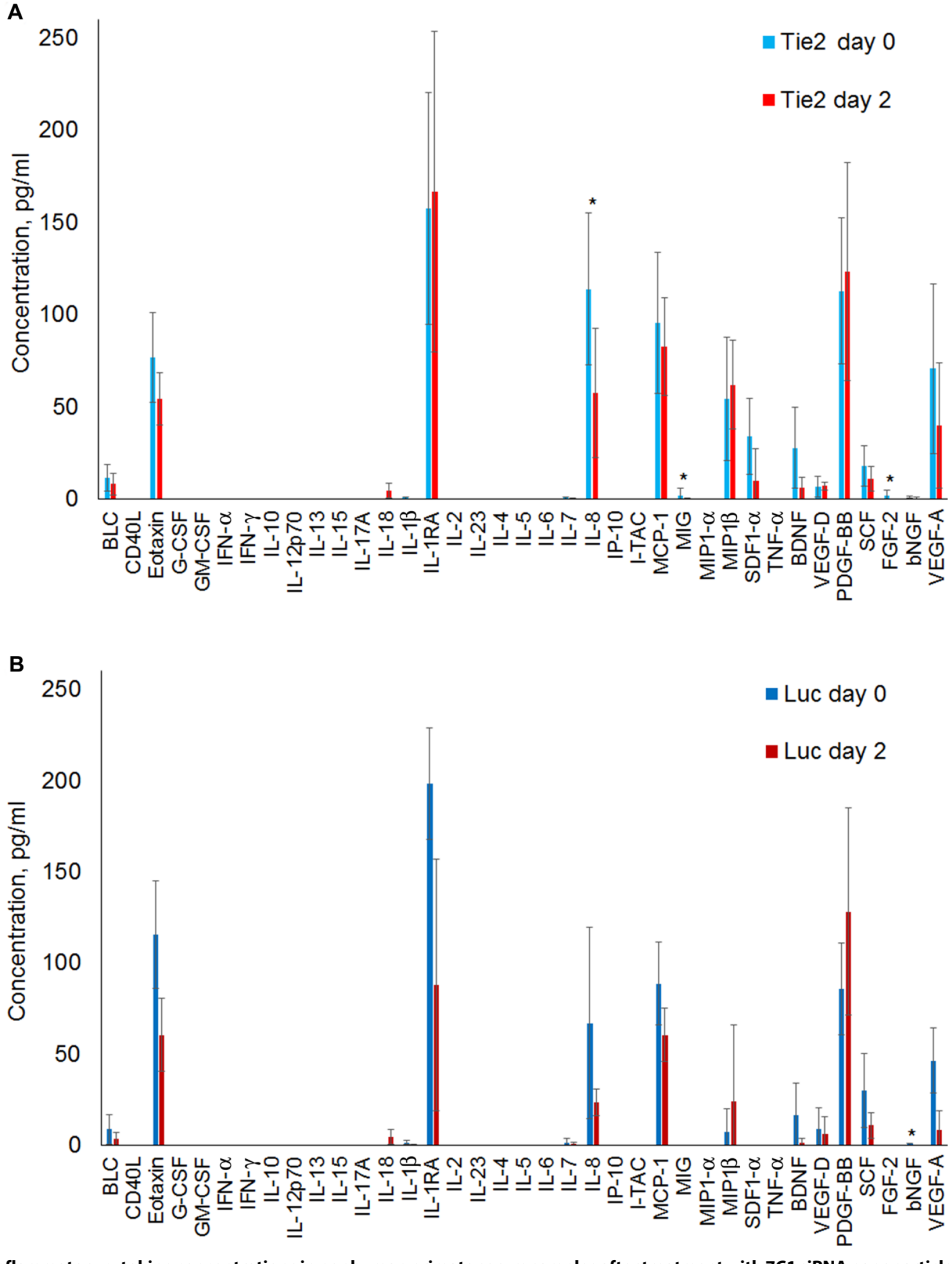
Changes in inflammatory cytokine concentrations in nonhuman primate serum samples after treatment with 7C1 siRNA nanoparticles. (**A**) Treatment with Tie2 siRNA. (**B**) Treatment with Luc control siRNA. Statistically significant differences (*P* < 0.05, *t* test) are denoted by the * symbol. G-CSF, granulocyte colony-stimulating factor; GM-CSF, granulocyte-macrophage colony-stimulating factor; IFN-α, interferon-α; TNF-α, tumor necrosis factor–α. BLC, brain-derived neurotrophic factor; I-TAC, IFN-inducible T cell alpha chemoattractant; MCP-1, monocyte chemoattractant protein-1; MIP1-α, macrophage inflammatory protein-α; BNDF, brain-derived neurotrophic factor; PDGF-BB, platelet derived growth factor-BB.

To investigate the inflammatory response to the 7C1 siRNA nanoparticles, we measured the serum concentrations of 37 inflammatory cytokines before and after 7C1 nanoparticle administration using a paired study experimental design (refer to Fig. 3). For nonhuman primates treated with 7C1-Tie2 siRNA nanoparticles, 3 of 37 inflammatory cytokines showed statistically significant changes in their mean concentrations (Fig. 3A). Specifically, interleukin-8 (IL-8) (4.35 to 0.00 pg/ml; *P* = 0.01, *t* test), monokine induced by IFN-γ (MIG) (2.15 to 0.19 pg/ml; *P* = 0.034, *t* test), and fibroblast growth factor–2 (FGF-2) (1.75 to 0.00 pg/ml; *P* = 0.024, *t* test) all decreased. However, the changes observed with Tie2 siRNA treatments were not observed in Luc control siRNA treatments (Fig. 3B). For the nonhuman primates treated with 7C1-Luc control siRNA nanoparticles, the only observed difference was a decrease in the mean β nerve growth factor (bNGF) concentration (0.49 to 0.00 pg/ml; *P* = 0.02, *t* test).

### Histopathology

**Fig. 4:**
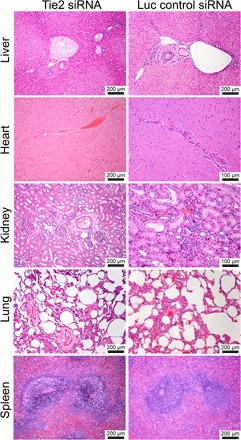
Representative posttreatment histology images from a subset of nonhuman primate tissues. Hematoxylin and eosin were used to stain the tissue. No deleterious effects, such as hepatocellular and cholestatic injury, were observed at this day 2 time point.

Complete necropsy was performed on the nonhuman primates 48 hours after treatment. We used these data to provide comprehensive histopathological interpretations on the effects caused by 7C1 siRNA nanoparticle usage. All nonhuman primates were found to be in good body condition and good postmortem preservation. No gross lesions, underlying pathologies, or aberrant vasculature was observed. Figure 4 shows representative histology images from several organs.

## DISCUSSION

7C1 nanoparticles delivering siRNA or microRNA have been deployed to achieve therapeutic effects in many rodent models of disease that involve inflammation and cancer (*13*, *15*–*21*). Building upon this work, this bridging study is designed to evaluate efficacy in more human-relevant animals. Although the animal numbers are limited (*n* = 4 for 7C1-Tie2 siRNA treatment groups and *n* = 3 for 7C1-Luc siRNA control groups) and Tie2 is not necessarily a clinically relevant therapeutic target, this remains a foundational study to aid a potential investigational new drug application. In terms of predictive value, demonstrating siRNA nanoparticle efficacy in nonhuman primates is an important step toward this goal. For example, Alnylam Pharmaceuticals recently completed a successful APOLLO phase 3 study of their liver-targeting siRNA nanoparticle patisiran after early studies in cynomolgus macaques, the same nonhuman primates used in this study (*8*–*10*).

Previous nonhuman primate studies have reported the ability of a liposomal siRNA formulation, Atu027, to elicit RNAi-mediated silencing in the lung endothelium of nonhuman primates after five doses (*12*). This report shows that the 7C1 formulated with siRNA can silence the endothelium of multiple organs with a single dose. We observed the most pronounced Tie2 mRNA silencing in the lung and the heart, followed by the retina, kidneys, pancreas, and bone (Fig. 1B). The high efficacy of Tie2 knockdown in the lungs, heart, and kidneys was consistent with the dose-response study in mice (fig. S1A). This effect, which is conserved across species, may indicate preferential interaction or access of the 7C1 nanoparticles with the vasculature of these organs. Beyond the use of RNAi to treat vascular diseases in these organs (*17*, *19*), 7C1 siRNA nanoparticles may be used to inhibit the region-specific angiogenesis associated with primary and metastatic tumors. For example, pancreatic and lung and bronchus cancer both have low 10-year survival rates (4.5 and 11%, respectively) (*31*); thus, the ability to combat cancer-associated angiogenesis in these organs can add a much needed treatment modality to improve patient outcomes. These types of anti-angiogenic interventions have already been successfully explored in several rodent models of cancer to great effect (*13*, *15*).

The gene knockdown observed in the retina is of particular interest, as it can potentially enable systemic RNAi treatments for diseases with an underlying vascular pathology that extends into the retinal vasculature, such as diabetic retinopathy (*32*). Another hypothesized potential use would be for retinal vessel occlusion therapy. New combination treatments may, for example, use RNAi alongside standard treatments that include isovolemic hemodilution, ocular massage, topical β-blocker, and intravenous acetazolamide (*33*).

Furthermore, at the evaluated dose, the 7C1-Tie2 siRNA formulation did not elicit significant gene knockdown in the liver endothelium (Fig. 1B). This property appears to be conserved across mice and cynomolgus macaques, as demonstrated in fig. S1A and in (*13*). In addition, this effect appears to be independent of the gene target; a similar lack of knockdown was observed in the liver of mice when using VE-cadherin siRNA (*13*). While the exact mechanism of action is currently unknown, these differences may be related to the endothelium’s phenotypic heterogeneity across organs (*34*). For example, liver endothelium is distinct with its fenestrations, and liver sinusoidal endothelial cells have special scavenger functions. Ultimately, this property may be useful for avoiding off-target effects that could be associated with delivery to the liver endothelium.

Because 7C1-Tie2 siRNA treatments resulted in an efficient Tie2 mRNA silencing in multiple organs after 48 hours, we also expected a concomitant decrease in endothelial cell Tie2 protein expression. However, the observed interanimal variability may suggest that the 48-hour time point was not optimal, and more time may be required before significant and robust protein decreases can be measured. Target protein levels can persist after mRNA transcript silencing based on their stability and rate of turnover (duration) within the cell (*35*, *36*). Ideally, protein levels would be examined in a time course; however, because of the terminal design of this study and the humane need to minimize invasive tissue harvest procedures, it was not possible to investigate multiple time points. As shown by flow cytometry analysis (Fig. 1C), most of the animals treated with Tie2 siRNA (three of four) showed a decrease in the percentage of Tie2^+^ endothelial cells among analyzed tissues, with the exception of the liver (two of four). The observed variability in Tie2 protein levels can also potentially be compounded by the heterogeneity of endothelial cells within tissues and across vascular beds that may not be reflected by the flow cytometry analysis (*37*–*40*). In addition, in the spleen, we observed a large of amount of Tie2 mRNA transcripts after Tie2 siRNA treatment, which contrasts with the low percentage of CD31^+^ cells that stained positive for Tie2 protein. The cause of this disconnect is unknown, although it may be related to sample preparation, as the spleen cell lysates used to measure Tie2 mRNA were more viscous than the other organs.

We performed blood chemistry panels on nonhuman primates before and after treatment for each type of siRNA to determine whether any large, acute events that disrupted normal liver and muscle function occurred (Fig. 2). After 48 hours, increases in ALK phosphatase following both Tie2 and Luc control siRNA treatments were observed, although the new values still remained within the healthy normal reference range (100.00 to 277.00 IU/liter). In addition, albumin showed a small but statistically significant decrease after Tie2 siRNA treatment; however, no albumin decrease was found with Luc control siRNA. Notably, the lower albumin level was still within the healthy reference range of 3.10 to 5.30 g/dl. In general, the absence of abnormal blood chemistry readings under the experimental conditions reported herein shows the potential tolerability and safety of 7C1. 7C1 did not detrimentally affect any liver functions, as measured by ALK phosphatase, ALT, AST, GGT, albumin, and total bilirubin concentrations. Furthermore, we observed no muscle damage, as measured by CK levels. While these data show no abnormalities after a single dose, some chronic conditions may require multiple doses over longer periods of time. Thus, future work must examine the blood chemistry after multiple 7C1 doses to better understand the long-term tolerability.

To perform a more in-depth discovery of any acute changes caused by the administration of 7C1 siRNA nanoparticles, we carried out an inflammatory cytokine screen on blood serum samples before treatment and 48 hours after treatment (Fig. 3). For nonhuman primates treated with 7C1-Tie2 siRNA nanoparticles, IL-8 increased, and both MIG and FGF-2 decreased. Because these changes could have been caused by the 7C1 material, the choice of siRNA, or both, we performed the same analysis using a different siRNA to control for siRNA effects. For the 7C1-Luc control siRNA treatments, the only change observed was a small decrease in mean bNGF concentration. Because the 7C1 material remained constant across both treatment conditions, the observed changes in inflammatory cytokine concentrations may be attributed to the downstream effects of siRNA-induced RNAi rather than the 7C1 formulation itself. To further expand this analysis, future work should explore cytokine concentration profiles at earlier time points, as well as after multiple doses.

To further confirm the encouraging blood chemistry measurements, liver function tests, and cytokine screen tolerability data, we performed full animal necropsies and histopathological analyses on the nonhuman primates and their tissues. No gross lesions or significant underlying pathologies were observed (Fig. 4), which supports the assertion that 7C1 is a well-tolerated material in nonhuman primates. This tolerability also recapitulates previous observations made in multiple rodent models (*13*, *15*–*21*).

In conclusion, early translational studies in nonhuman primates have shown that nanoparticles made from the ionizable, low–molecular weight polymer 7C1 and siRNAs can induce RNAi in the endothelium of multiple organs. The greatest amount of gene knockdown was observed in the lung and heart, which is in good agreement with previous rodent studies (*13*, *16*, *17*, *19*, *21*, *41*). In addition, effective gene knockdown was seen in the vasculature of the retina, kidney, pancreas, and bone, which has the potential to create new vasculature-focused, RNAi-based treatments for these organs and tissues. In addition, at a dose of 1 mg/kg, no statistically significant knockdown was observed in the liver endothelium, which may aid in the avoidance of liver-associated off-target effects. Furthermore, we found no acute signs of toxicity over the time course of these studies. To our knowledge, and given the above data, this study provides novel evidence of endothelial gene silencing in large nonhuman primates using systemically administered siRNA nanoparticles.

## MATERIALS AND METHODS

### Material synthesis

7C1 was produced by reacting polyethyleneimine (PEI; *M*_n_ = 600) with 2-tridecyloxirane. 2-tridecyloxirane was synthesized as previously described (*42*) by the dropwise addition of 1-pentadecene (Tokyo Chemical Industry Co., Ltd.) to a 2× molar excess of 3-chloroperbenzoic acid (Sigma-Aldrich) in dichloromethane (BDH Chemicals) under constant stirring at room temperature. After reacting for 8 hours, the reaction mixture was washed three times with equal volumes of a supersaturated aqueous sodium thiosulfate solution (Sigma-Aldrich). After each wash, the organic layer was collected using a separation funnel. Similarly, the organic layer was then washed three times with 1 M NaOH (Sigma-Aldrich). Anhydrous sodium sulfate was added to the organic phase and stirred overnight to remove any remaining water. The organic layer was concentrated under vacuum to produce a slightly yellow, transparent oily liquid. This liquid was vacuum-distilled (~6.5 Pa, ~80°C) to produce clear, colorless 2-tridecyloxirane. PEI with a molecular weight number of 600 (PEI600, Sigma-Aldrich) was combined with 200 proof ethanol and 2-tridecyloxirane at an expoxide/PEI molar ratio of 14:1. The mixture was heated at 90°C for 48 hours with constant stirring in the dark. The crude product was mounted on a Celite 545 (VWR International) precolumn and purified via flash chromatography using a CombiFlash Rf machine with a RediSep Gold Resolution silica column (Teledyne ISCO) with gradient elution from 100% CH_2_Cl_2_ to 75:22:3 CH_2_Cl_2_/MeOH/NH_4_OH_aq_ (by volume) over 40 min. Thin-layer chromatography was used to test the eluted fractions for the presence of product using an 87.5:11:1.5 CH_2_Cl_2_/MeOH/NH_4_OH_aq_ (by volume) solvent system. Fractions containing the product were combined, dried under ramping high vacuum for 12 hours, and stored under a dry, inert atmosphere until used. All siRNAs were synthesized by Alnylam Pharmaceuticals, as described previously, and characterized by electrospray mass spectrometry, and anion exchange high-performance liquid chromatography (*43*).

### Nanoparticle formulation

Nanoparticles were formulated using a microfluidic mixing device, as described previously (*42*, *44*, *45*). Briefly, 7C1 and 1,2-dimyristoyl-*sn*-glycero-3-phosphoethanolamine-*N*-[methoxy(polyethylene glycol)-2000] (Avanti Polar Lipids) were combined in 200 proof ethanol. RNA was diluted with UltraPure, DNase/RNase-Free, endotoxin-free distilled water (Invitrogen) and sterile 100 mM (pH 3.0) QB Citrate Buffer (Teknova Inc.) to a final citrate concentration of 10 mM. The ethanol and citrate streams were loaded into gastight glass syringes (Hamilton Co.), and using a microfluidic mixing device, the ethanol and citrate streams were combined and mixed in a 1:3 volumetric flow rate ratio (combined total flow rate equal to 2.4 ml/min) to produce nanoparticles. Using glassware washed for 24 hours in 1.0 M NaOH (Sigma-Aldrich) for endotoxin removal and sterilized in a steam autoclave, nanoparticles were dialyzed against sterile, endotoxin-free PBS using 20,000 molecular weight cutoff Slide-A-Lyzer G2 dialysis cassettes. Dialyzed nanoparticles were sterile-filtered using 0.2-μm poly(ether sulfone) filters (Genesee Scientific) and characterized with a Zetasizer NanoZS machine (Malvern). The concentration of RNA was determined by theoretical mass balance calculations and confirmed by NanoDrop measurement (Thermo Fisher Scientific). The final nanoparticles contained a 1:0.19:5 mass ratio of 7C1 to 1,2-dimyristoyl-*sn*-glycero-3-phosphoethanolamine-*N*-[methoxy(polyethylene glycol)-2000] to RNA.

### Animal studies

All animal studies were performed at the Massachusetts Institute of Technology (MIT), Division of Comparative Medicine. All procedures and studies conducted were approved by the MIT Institutional Animal Care and Use Committee and were consistent with all applicable local, state, and federal regulations.

For rodent work, 8-week-old female C57BL/6 mice were used. All mice received nanoparticles (10 μl/g) via tail vein injection. For nonhuman primates, 5-year-old female cynomolgus monkeys (*Macaca fascicularis*) were used, and nanoparticles were warmed to 37°C immediately before a 15-min intravenous infusion (5 ml/kg) via the cephalic vein. For the nonhuman primates portion of this study, a paired experimental design was used for blood chemistry and inflammatory cytokine analysis, where pre- and posttreatment levels were compared.

For nonhuman primate mRNA transcript and protein expression work, comparisons were made between 7C1-Tie2 siRNA and 7C1-Luc siRNA treatments. An untreated control group was not used to comply with institutional animal welfare and humane use directives.

### Tie2 gene silencing

Tie2 or Luc control siRNAs were used in these studies. The sequences and modification of the siRNAs were as follows: GAAGAuGcAGuGAuuuAcAdTsdT (Tie2 siRNA sense), UGuAAAUcACUGcAUCUUCdTsdT (Tie2 siRNA antisense), cuuAcGcuGAGuAcuucGAdTsdT (Luc control sense), and UCGAAGuACUcAGCGuAAGdTsdT (Luc control antisense). The lowercase letters correspond to nucleotides modified with 2-*O*-methyl modifications, which decrease immunostimulation and promote antisense strand selection in the RNA-induced silencing complex.

Gene silencing was examined 48 hours after intravenous injection. For mouse studies, a minimum of five animals were used per treatment group. Mice were euthanized by CO_2_ asphyxiation. For nonhuman primate studies, four animals received Tie2 siRNA and three received Luc control siRNA. Nonhuman primates were euthanized via intravenous administration of pentobarbitone. For both species, a secondary euthanasia method was used. Immediately after euthanasia, necropsy was performed, and tissues were harvested. Each organ was divided into halves. Half of the organ was fixed for histopathology. The other half was snapped frozen in liquid nitrogen and stored at −80°C until the mRNA transcripts quantification.

### Quantification of mRNA transcripts

Frozen tissues were pulverized to form a powder, and tissue lysates were prepared in Tissue and Cell Lysis Buffer (Epicentre) supplemented with proteinase K (0.5 mg/ml; Epicentre). The mixture was agitated at 1400 rpm for 2 hours at 65°C and centrifuged at 16,000*g* to remove any debris. The mRNA levels in the supernatant (lysate) were quantified using the QuantiGene 2.0 luminescent-based branched DNA Assay Kit and QuantiGene 2.0 probes against Tie2 and glyceraldehyde-3-phosphate dehydrogenase (Gapdh; Affymetrix) according to the manufacturer’s protocol. Luminescent signal was measured with a Tecan Infinite 200 PRO plate reader. To avoid signal saturation and to ensure that all luminescent signals remained within their linear regions, a standard curve for each tissue and target gene was constructed using samples from PBS-treated mice to determine the optimal dilutions for assay samples. The relative silencing in treated groups was determined by calculating the ratio of target gene luminescence to *Gapdh* housekeeper gene luminescence. All values were normalized to the target/housekeeper gene ratio from PBS-treated mice. To graph the data, the mean values of the three 7C1-Luc siRNA treatments were used to normalize each of the four 7C1-Tie2 siRNA treatment values. The mean normalized 7C1-Tie2 siRNA treatment values were then plotted along with their SD.

### Tissue dissociation, FACS analysis, and CD31^+^cell enrichment

Single-cell suspensions of all 13 freshly excised nonhuman primate tissues were prepared using a gentleMACS Dissociator (Miltenyi Biotec) according to the manufacturer’s protocols and a previously published methodology (*46*), depending on the specified tissue. Single-cell suspensions were prepared in a passive PBS-EDTA buffer dissociation buffer [1× PBS (pH 7.2) 0.5% bovine serum albumin, and 2 mM EDTA], and suspensions were passed through 70-μm filters (catalog no. 22363548, Thermo Fisher Scientific). All tissue and material sample–derived, single-cell populations were then subjected to red blood cell lysis with 5 ml of 1× red blood cells lysis buffer (catalog no. 00-4333, eBioscience) for 5 min at 4°C. The reaction was terminated by the addition of 20 ml of sterile 1× PBS. The remaining cells were centrifuged at 300 to 400*g* at 4°C and resuspended in a minimal volume (~50 μl) of the eBioscience Staining Buffer (catalog no. 00-4222) for antibody incubation for fluorescence-activated cell sorting (FACS). For FACS, all samples were then costained in the dark for 25 min at 4°C with two of the fluorescently tagged monoclonal antibodies specific for the cell markers CD31 [5 μl (2 μg) per sample; anti-human CD31–Alexa Fluor 488, Clone WM59, catalog no. 303110, BioLegend] and CD202b (Tie2/Tek) [5 μl (2 μg) per sample; anti-human Tie2–Alexa Fluor 647, Clone 33.1 (Ab33), catalog no. 334210, BioLegend]. Two milliliters of the eBioscience Flow Cytometry Staining Buffer (catalog no. 00-4222, eBioscience) was then added, and the samples were centrifuged at 400 to 500*g* for 5 min at 4°C. Supernatants were removed by aspiration, and this wash step was repeated two more times with staining buffer. Following the third wash, each sample was resuspended in 500 μl of the Flow Cytometry Staining Buffer and run through a 40-μm filter (catalog no. 22363547, Thermo Fisher Scientific) for eventual FACS analysis using a BD LSR II (BD Biosciences). For proper background and laser intensity settings, unstained, single antibody, and immunoglobulin G (labeled with either Alexa Fluor 488 or Alexa Fluor 647, BioLegend) controls were also analyzed. To graph the data, the mean values of the three 7C1-Luc siRNA treatments were used to normalize each of the four 7C1-Tie2 siRNA treatment values. The mean-normalized 7C1-Tie2 siRNA treatment values were then plotted along with their SD.

### Cytokine screen

Pre- and posttreatment serum samples from nonhuman primates were collected. The concentration of inflammatory cytokines in these samples was sent to Affymetrix eBioscience for the ProcartaPlex nonhuman primate 37-plex inflammatory cytokine screen service. To graph the data, the mean values of the three 7C1-Luc siRNA treatments at days 0 (before treatment) and 2 (after treatment) were plotted along with their SD. Similarly, the mean values of the three 7C1-Tie2 siRNA treatments at days 0 (before treatment) and 2 (after treatment) were plotted along with their SD.

### Statistical analysis

Statistical analyses were performed using GraphPad Prism software (GraphPad Software, Inc.). Results are depicted as mean ± SD, and statistical tests are indicated in the figures. Differences were considered statistically significant if *P* < 0.05.

## Supplementary Material

http://advances.sciencemag.org/cgi/content/full/4/6/eaar8409/DC1

## SUPPLEMENTARY MATERIALS

Supplementary material for this article is available at http://advances.sciencemag.org/cgi/content/full/4/6/eaar8409/DC1 fig. S1. 7C1 validation and characterization. fig. S2. Flow cytometry analysis.
