# Supplementary material for: Endothelial siRNA delivery in nonhuman primates using ionizable low–molecular weight polymeric nanoparticles
Source: Sci Adv. 2018 Jun 27;4(6):eaar8409. doi: 10.1126/sciadv.aar8409 (PMC6021147; doi:10.1126/sciadv.aar8409)
Supplement: http://advances.sciencemag.org/cgi/content/full/4/6/eaar8409/DC1 [file supp_4_6_eaar8409__index.html]

Science Advances | Science Advances

## Supplementary Materials

**This PDF file includes:**

- fig. S1. 7C1 validation and characterization.
- fig. S2. Flow cytometry analysis.

Download PDF

**Files in this Data Supplement:**

- Adobe PDF - aar8409\_SM.pdf
