# Supplementary material for: Endothelial siRNA delivery in nonhuman primates using ionizable low–molecular weight polymeric nanoparticles
Source: Sci Adv. 2018 Jun 27;4(6):eaar8409. doi: 10.1126/sciadv.aar8409 (PMC6021147; doi:10.1126/sciadv.aar8409)
Supplement: http://advances.sciencemag.org/cgi/content/full/4/6/eaar8409/DC1 [file aar8409_SM.pdf]

Science Advances

AAAS

advances.sciencemag.org/cgi/content/full/4/6/eaar8409/DC1

# Supplementary Materials for

## Endothelial siRNA delivery in nonhuman primates using ionizable low–molecular weight polymeric nanoparticles

Omar F. Khan, Piotr S. Kowalski, Joshua C. Doloff, Jonathan K. Tsosie, Vasudevan Bakthavatchalu, Caroline Bodi Winn, Jennifer Haupt, Morgan Jamiel, Robert Langer, Daniel G. Anderson

Published 27 June 2018, *Sci. Adv.* **4**, eaar8409 (2018)
DOI: 10.1126/sciadv.aar8409

**This PDF file includes:**

- fig. S1. 7C1 validation and characterization.
- fig. S2. Flow cytometry analysis.

**Supplementary Materials**

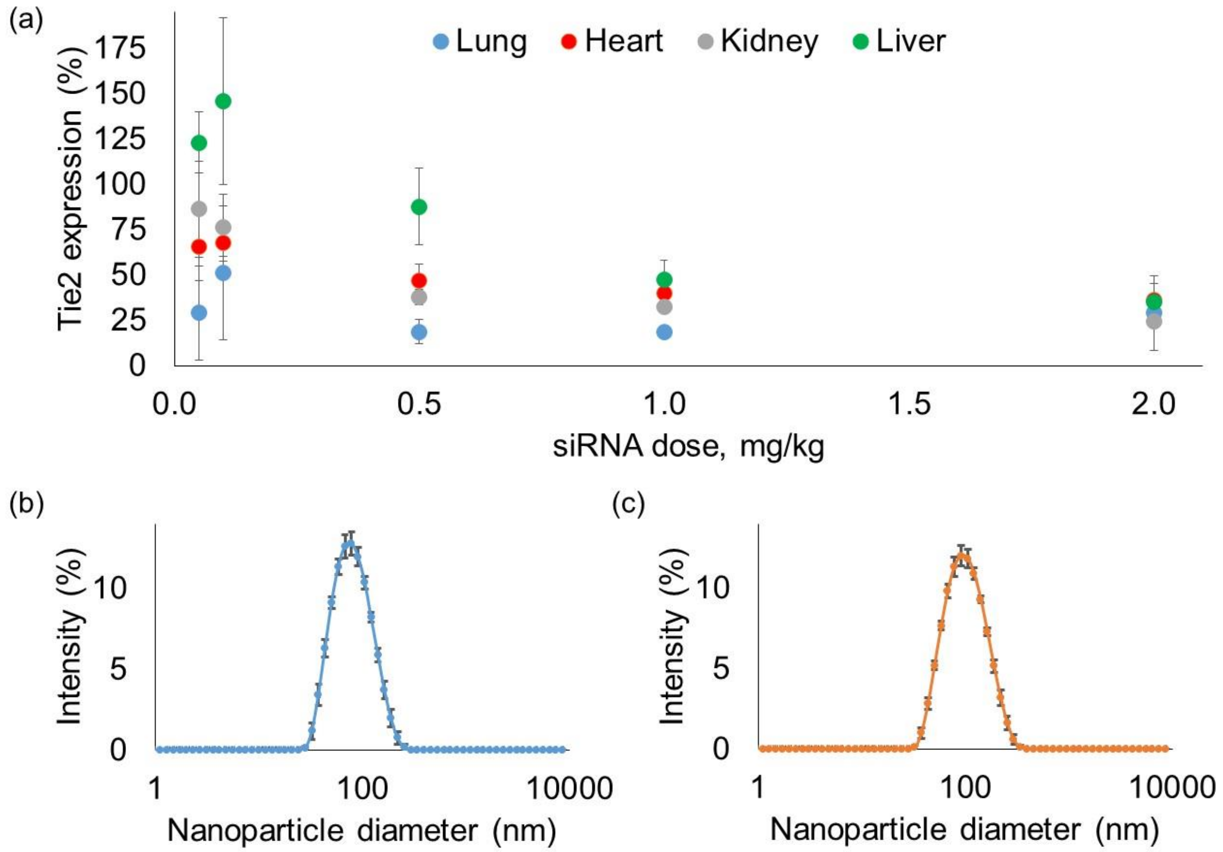


**fig. S1. 7C1 validation and characterization.** (**A**) 7C1 and Tie2 siRNA QA/QC. Prior to testing in nonhuman primates, the 7C1 lot and Tie2 siRNA were both tested in mice to confirm gene knockdown ability in the vascular beds of the lung, heart, kidney and liver. This data was also used to inform the choice of dose in nonhuman primates. N = 5 and error bars are ± S.D. (**B**) 7C1 nanoparticle size distribution for Tie2 siRNA nanoparticles. N = 3 and error bars are ± S.D. (**C**) 7C1 nanoparticle size distribution for Luc control siRNA nanoparticles. N = 3 and error bars are ± S.D.

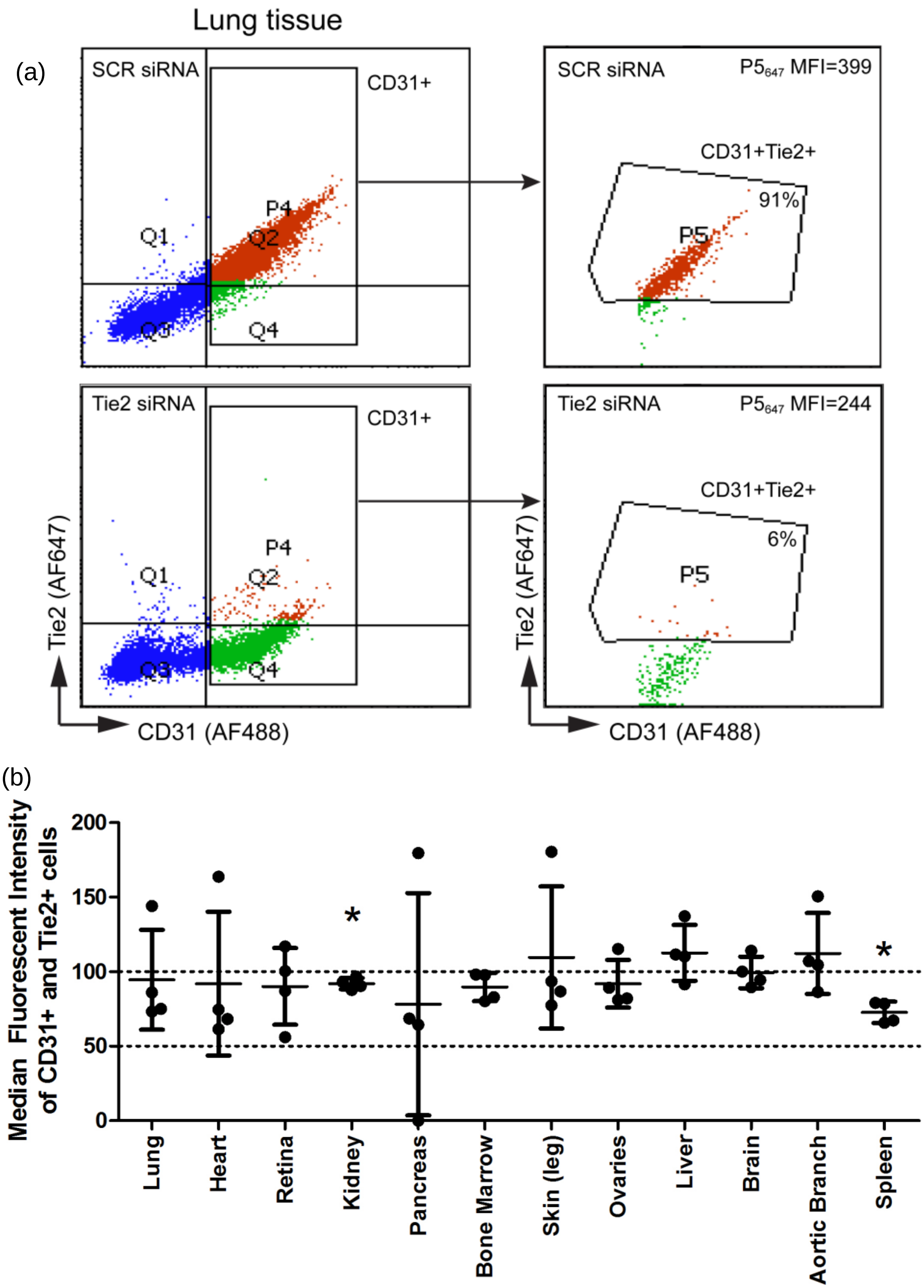


**fig. S2. Flow cytometry analysis.** (**A**) Example gating strategy and quantification of the Tie2 protein expression in tissue single cell subsections by flow cytometry. Tissues were minced and

dissociated into single cells suspensions using a Gentle MACS dissociator (Miltenyi). Prior to staining, samples were filtered through 70 µm pore filters and red blood cells were lysed with RBC lysis buffer (BioLegend). Single cell suspensions were stained with anti-human CD31 AF488 (clone WM59) and anti-human Tie2 AF647 (clone 33.1) and suspended in PBS with 0.5% PFA prior to analysis. Samples were analyzed on FACS LSR II HTS-2 flow cytometer (BD) running BD FACSDIVA software. Analysis gates were set using unstained and single stain cell suspensions. (**B**) The Median Fluorescent Intensity (MFI) of Tie2-expressing CD31+ (endothelial) cells. Error bars are ± S.D. and * indicates a significant change ($p < 0.05$, t-test) in Tie2 protein levels after Tie2 siRNA treatment, as compared to Luc control siRNA treatments. Note that organs along the x-axis are arranged to match the order found in **Fig. 1**b and c.
